# Joining the National Resident Matching Program Fellowship Match: the hematopathology experience

**DOI:** 10.1016/j.acpath.2025.100230

**Published:** 2025-12-12

**Authors:** Elizabeth L. Courville, Robert Seifert, Sam Sadigh, Xueyan Chen, Jeanette L. Calli, Robert Hasserjian, Alexa J. Siddon

**Affiliations:** a Department of Pathology, University of Virginia, Charlottesville, VA, USA; b Department of Pathology, Immunology, and Laboratory Medicine, University of Florida College of Medicine, Gainesville, FL, USA; c Department of Pathology, Brigham and Women's Hospital and Harvard Medical School, Boston, MA, USA; d Translational Science and Therapeutics Division, Fred Hutchinson Cancer Center, Department of Laboratory Medicine and Pathology, University of Washington, Seattle, WA, USA; e National Resident Matching Program, Washington, DC, USA; f Department of Pathology, Massachusetts General Hospital and Harvard Medical School, Boston, MA, USA; g Departments of Laboratory Medicine and Pathology, Yale School of Medicine, New Haven, CT, USA

**Keywords:** Fellowship, Hematopathology, Match, National Resident Matching Program, NRMP, Pathology, Specialties Matching Service, SMS

## Abstract

Hematopathology fellowships are a critical subspecialty within the field of pathology, and with a progressively earlier and earlier fellowship interview timeline, hematopathology decided to join the Pathology Fellowship Match in 2025. The pros and cons of participating in the National Resident Matching Program Match were evaluated, and ultimately it was decided to be undertaken. The National Resident Matching Program requires that 75% of programs and fellowship positions should be entered in order to sponsor the Match. The Society for Hematopathology Education Committee lead the process to recruit hematopathology fellowships into the Match, initiating a survey to assess initial interest, creating a listserv for program director questions, and maintaining a website with programs that ultimately decided to participate. The initial survey results showed that 79% of hematopathology program directors responded “yes” or “maybe” to whether they would participate in a formal Match. The Society for Hematopathology Education Committee then proceeded with a memorandum of understanding to show commitment to the Match. Multiple efforts to disseminate information followed, including informational webinars, social media outreach, and emails to program directors. Ultimately 83% of hematopathology fellowships participated in the 2025 Match, with 85% of positions filling, and 94.7% of hematopathology applicants matching. A follow-up program director survey showed that 98% of respondents planned to participate in the 2026 Match. This feedback solidifies that the Pathology Fellowship Match has shown mutual benefit for programs and applicants.

In 2011, the Council of the Association of Pathology Chairs (APC) published its recommendation to implement a pathology subspecialty fellowship matching program.^1^^,^^2^ More than 10 years later, Forensic Pathology initiated the inaugural Pathology Fellowship Match (2023 Match for the 2024 appointment year) through the National Resident Matching Program (NRMP) and supported by the National Association for Medical Examiners (NAME). Forensic Pathology was again the only pathology subspecialty in the 2024 Match for the 2025 appointment year.

The process of fellowship application and selection is complicated and nuanced with multiple stakeholders. As such, it has been the subject of much discussion within the field of pathology as evidenced by literature on the topic.^3^, ^4^, ^5^, ^6^, ^7^ The benefits to the Pathology Fellowship Match include the organization of a unified process that has streamlined dates nationally, allows applicants to explore all potential fellowship locations/hospitals, and allows programs to interview numerous applicants without offering time-limited offers to candidates. Potential cons to a Match are that the majority of programs are required to participate, the likely increased numbers of interviews per spot programs generally participate in, and the costs associated with the Match. In the past, hematopathology fellowships had attempted an agreed-upon, or “unified” timeline for interviewing and offering fellowship positions, but it was not enforceable and ultimately failed due to noncompliance. An updated version of the unified timeline was considered, but ultimately it felt that it would not be successful.

In this article, we present the hematopathology (HP) subspecialty experience in joining the NRMP Fellowship Match, first participating in the 2025 Match for the 2026 appointment year. We discuss the key communication efforts involved, summarize the HP Match results, and present findings of the HP program directors’ (PD) pre- and post-Match surveys. Finally, we introduce guidance for future fellowship application/selection cycles. A timeline summary is presented in Table 1.

**Table 1:** Hematopathology fellowship match timeline.

	Action	Details
2020	Preliminary survey sent to HP PD listserv to gauge interest in the MMatch.	Yes 19 (22%)
		Maybe 11 (13%)
	**Question**: Would your program participate in a HP fellowship Match if available?	No 8 (9%)
		No response 48 (56%)
2023 HP PD meeting (March 2023)	Discussion about the possibility of HP participation in fellowship MMatch vs a unified timeline	Invited panellists representing fellowships using the Common Timeline, and Ms. Jeanette Calli representing the NRMP.
	**Takeaway:** Match would be preferred	
Spring/Summer/Fall 2023	Multiple emails regarding the Match and inviting commitment to participate/sign memorandum of understanding (MOU) – Siddon/Courville/Hasserjian	
	The SH-EC hosted virtual Q&A session with Jeanette Calli (NRMP representative) and Alexa Siddon	
January 2, 2024	Decision made and SH sponsor agreement signed	68/86 (78%) of programs
January/February 2024	SH Hematopathology Fellowship website updated to include information about the match.	
	FAQ documents prepared (Seifert/Sadigh)	
	Match commitment publicized (SH Communications committee)	
2024 HP PD meeting (March 2024)	Presented MMatch process and data and brief Q&A (Courville)	
March–December 2024	Gathered additional information from participating programs (to facilitate 1st year process with NRMP)	71/86 = 83% of programs; 113/151 (85% of positions) – final list of participating programs submitted to NRMP
	Welcomed additional programs to join	
	Continued to provide trainees/programs with information (including virtual Q&A session)	
	Served as a resource for other pathology fellowship programs considering joining the Pathology MMatch	
July 2024	Introduction to the Pathology Fellowship MMatch, ASCP webinar	
January 1, 2025	Programs started interviewing	
April 15, 2025	Rank Lists Due	
April 30, 2025	Match day	

ASCP: American Society for Clinical Pathology; HP: hematopathology; NRMP: National Resident Matching Program; PD: program director; SH-EC, Society for Hematopathology Education Committee.

## Process of establishing the hematopathology Match

**Fig. 1:**
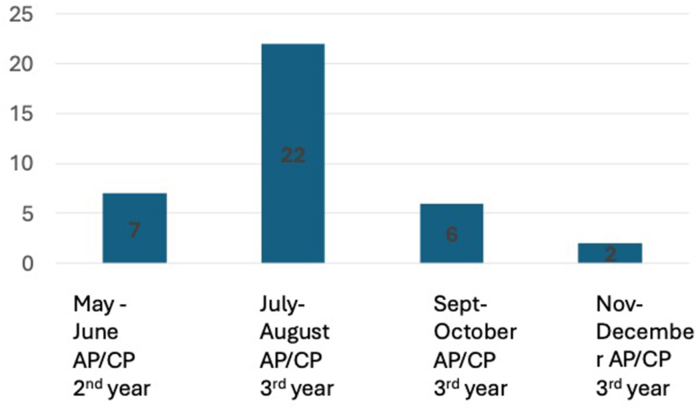
Pre-Match program director survey response to question: When does your program start interviewing fellowship applicants?

For a subspecialty to join the NRMP's Specialties Matching Service (SMS), a sponsoring organization must affirm that a sufficient percentage of specialty fellowship programs and positions have agreed to actively participate in the Match and that a sufficient percentage of available positions for the appointment year will be registered for the Match; specifically, 75% of programs and 75% of fellowship positions should be entered into the Match. The Society for Hematopathology (SH) was the sponsoring organization for the subspecialty of HP, with the Society for Hematopathology Education Committee (SH-EC) leading the process. The reason for this 75% minimal participation rate is that mathematically matches are more successful when the majority of programs participate; therefore, the NRMP has enforced this criteria to ensure a rewarding outcome for both programs and participants.^8^ In 2021, a listserv for HP PDs was created by the SH-EC, and an initial survey was distributed through the listserv to assess the interest level in joining the Match. The respondents shared that the majority of programs (59%) began interviewing applicants in July and August of the 3rd year of residency (assuming a 4-year residency), therefore about 24 months prior to the start of fellowship (Fig. 1). Further survey results showed that the 79% of HP PDs who responded said “yes” or “maybe” when asked if they would be interested in joining the NRMP Pathology Fellowship Match. Further group discussion by HP PDs at the 2023 annual HP PD meeting, including a representative from the NRMP, solidified sufficient interest to pursue formal agreements among HP fellowship programs to participate in the 2025 Match for 2026 appointment year. The SH-EC modeled their approach after that of the NAME and solicited a memorandum of understanding (MOU) from each program agreeing to participate in the Match between the months of March and December 2024. The MOU included a “no offers” commitment stating that the participating program would not make any offers to applicants for any of their positions for the 2026-27 fellowship year (normally such offers would have been extended in July–August 2024, based on the survey results of prior years). It also included was a commitment that participating programs would not interview candidates until January 1, 2025 (normally candidates would have been interviewed in May–June 2024).

From April 2023 through January 1, 2024, the SH-EC, supported by the SH-executive committee, exerted a concerted effort to reach all HP PDs and encourage participation in the Match by way of signing the MOU. This involved updating contact information for PDs, multiple rounds of emails to PDs, advertising to applicants via social media, reaching out personally to professional contacts, and hosting a virtual Q&A session sponsored by SH. The SH-EC developed a webpage on the SH website listing the HP fellowship programs committed to participating in the Match and included links to individual fellowship webpages.^9^ Understandably, there were many questions from PDs, program coordinators, and potential applicants regarding the Match. Some of the most common were regarding the timeline and cost of the Match and how to register. A common misconception was that the application process would be standardized (such as using the Electronic Residency Application Service [ERAS] system) along with the selection process. There were questions regarding how to prioritize internal applicants, how the Match would affect small programs, and how to handle military applicants, applicants pursuing multiple fellowships at the same institution, and two-year fellowship programs. The SH-EC developed two frequently asked questions (FAQ) documents, one for applicants and one for PDs/coordinators (see Supplemental Material 1 and Supplemental Material 2). After receiving signed MOU documents from 78% of fellowship programs, a decision was made at the beginning of January 2024 for HP to join the Match.

After the initial decision was made, an additional three programs committed to the Match (total of 83% of programs participating). As it was the first year HP was participating in the Match, the SH-EC helped gather contact information on the programs committed to the Match to facilitate the registration process with the NRMP. The SH-EC hosted a second virtual Q&A session for PDs and program coordinators on November 14, 2024. Hematopathology's participation in the Match was advertised via social media (in collaboration with the SH communications committee), by way of emails sent to the Association for Academic Pathology Residency Program Directors and Associate Residency Program Directors (PRODS) listserv and a residency program coordinators listserv, and at the SH companion meeting and fellowship fair hosted at the United States and Canadian Association of Pathologist (USCAP) annual meeting in March 2024. Representatives from the SH-EC were panel participants in a resident-directed American Society of Clinical Pathology (ASCP) webinar posted on ASCP Communities. The webinar included representatives from the subspecialties of Forensic Pathology, Molecular and Genetic Pathology, and Bone and Soft tissue Pathology, the latter two of which joined the Pathology Fellowship Match following HP's decision.

## Outcomes of the Match

The 2025 NRMP Pathology Fellowship Match for appointment year 2026 was successful for all included specialties, with 109 of the 155 (70.3%) certifying programs filling, 208 of the 264 (78.8%) positions filling, and 208 of the 222 (93.7%) applicants matching (Table 2).^10^ Specifically for hematopathology, 80% of the 70 certified programs filled all offered positions, with 85% of the 127 certified positions filled. Of the 114 applicants who preferred hematopathology (as defined by ranking the specialty first on their rank order list), 108 (94.7%) of the hematopathology applicants matched to a hematopathology program, and the remainder did not match to any pathology fellowship program. After Match Day, all programs in the Match with unfilled positions received a list of applicants who did not match. Similarly, applicants received a list of programs that did not fill. At that point, selection for the unfilled positions occurred outside of the NRMP Match and was not tracked by the NRMP.

**Table 2:** Overall pathology fellowship Match outcomes.

	All programs	Hematopathology	Bone & soft tissue	Forensics	Molecular genetics
Programs enrolled	158	71	13	40	34
Programs Certified	155	70	12	40	33
Programs Filled	109 (70.3%)	56 (80%)	9 (75%)	21 (52.5%)	23 (69.7%)
Programs Unfilled	46 (21.2%)	14 (20%)	3 (25%)	19 (47.5%)	10 (30.3%)
Positions Filled	208 (78.8 %)	108 (85%)	10 (76.9%)	49 (69%)	41 (77.4%)
Positions Unfilled	56 (21.2%)	19 (15%)	3 (23.1%)	22 (31%)	12 (22.6%)
Matched Applicants	208 (93.7%)	108 (94.7%)	10 (76.9%)	49 (96.1%)	41 (93.2%)
Unmatched Applicants	14 (6.3%)	6 (5.3%)	3 (23.1%)	2 (3.9%)	3 (6.8%)

Data were adapted from the NRMP Match Results Statistics. NRMP: National Resident Matching Program.

**Fig. 2:**
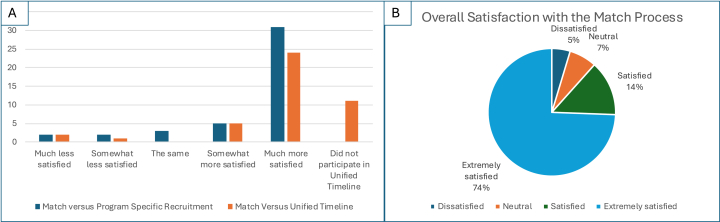
**A)** Post-Match survey asked about program satisfaction compared with other recruitment methods. **B)** Overall program director satisfaction with the Match process.

Following the conclusion of the Match in April 2025, the SH-EC distributed a survey to HP PDs via email with an invitation to participate in a survey via an online link. Forty-five of the 47 respondents (out of 86 total programs) had participated in the 2025 fellowship Match, and two programs had chosen not to participate in the Match. Thirty-three percent of respondents reported “about the same” number of HP fellowship applicants as in previous years while 63% received more. Respondents reported interviewing “about the same” (32%) or more (58%) applicants than in previous years, with only 7% reporting fewer applicant interviews. Reported satisfaction with the Match is presented in Fig. 2. All but one of the respondents (98%) noted they plan to participate in the 2026 Match (for 2027 academic year).

## Summary

**Fig. 3:**
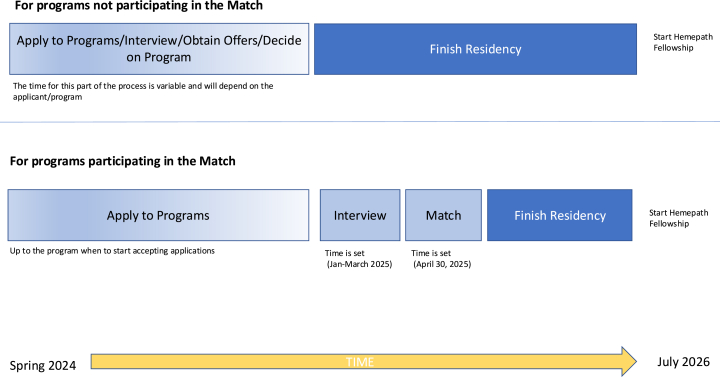
Comparison of timeline for programs participating in the match vs those not participating in the match.

These survey results underscore that the inaugural year for HP's participation in the Pathology Fellowship Match was highly successful. Gaining the momentum to join the Match was a multi-year process involving a concerted effort by multiple individuals and organizations. Extensive communication through multiple platforms (listserv, mass emails, direct emails, in person communication and presentations, webinars, virtual Q&A sessions, informational website, and FAQ documents) was vital to the success. Challenges for the SH-EC included the time and organization required to maintain contact with all the HP PDs, given that HP is a large subspecialty with many programs.^11^ For applicants, the Match process allows residents more time to fully explore pathology subspecialty options and solidify career goals by delaying the fellowship selection process by up to 18 months compared to the prior timeline (Fig. 3). An applicant participating in the Match is less likely to be pressured to accept a fellowship offer before having time to fully explore other options. Beneficial to both programs and applicants, the Match provides a more structured process for fellowship/fellow selection and professionalizes the recruitment process for the specialty. Our hope is that the anticipated benefits of HP participation in the fellowship Match become apparent to more programs in the months and years to come, increasing participation by both programs and applicants. The Match also allows for detailed data collection that can assist the HP training pipeline as has been done in other specialties.^12^^,^^13^ Future considerations include the incorporation of the ERAS into the application process. Hematopathology will be participating in the 2026 Match for the 2027 academic year.

## Funding

This research received no specific grant from any funding agency in the public, commercial, or not-for-profit sectors.

## Declaration of competing interest

The authors declare that they have no known competing financial interests or personal relationships that could have appeared to influence the work reported in this paper.
