# Supplementary material for: Joining the National Resident Matching Program Fellowship Match: the hematopathology experience
Source: Acad Pathol. 2025 Dec 12;13(1):100230. doi: 10.1016/j.acpath.2025.100230 (PMC12756700; doi:10.1016/j.acpath.2025.100230)
Supplement: Multimedia component 1 [file mmc1.docx]

**Supplemental Material 1**

Frequently Asked Questions: The NRMP Match for Hematopathology Fellowship

The selection process for prospective fellows in Hematopathology is switching to the NRMP Pathology Fellowship Match **for positions that begin in 2026**. This document assumes you are a program director/representative of a Hematopathology Fellowship program that has agreed to participate in the Match. Hematopathology will participate in a multi-specialty Pathology Fellowship Match with Forensic Pathology and possibly other subspecialties in Pathology who are considering a Match.

1. Now that we have agreed to participate in the Match, what happens next?

Below is an approximate timeline of events:

- 1. **Fall 2024**: applicants are encouraged to submit applications to their programs of your choice before **January 1, 2025.**
  2. You can begin interviewing applicants starting **January 2025**.
  3. **February 5^th^ 2025 at noon, ET: Pathology Fellowship Match opens for registration:** this is when your program signs a legally binding [Match Participation Agreement](https://www.nrmp.org/policies/) with the NRMP, and also when applicants can begin registering for the Match.
  4. **March 12, 2025 at noon, ET:** Ranking opens.
  5. **April 2, 2025 at 11:59 PM, ET:** Quota Change Deadline (Quota is the number of positions you program intends to fill in the Match. Programs must withdraw by the deadline or make any change to their number of positions in the Match).
  6. **April 16, 2025 at noon, ET**: Rank Order List Certification Deadline (for you and for applicants). Rank lists cannot be edited after this.
  7. **April 30, 2025 at noon, ET:** **Match day!**
  8. **July 2026:** your new fellow(s) start

1. Do we use ERAS to receive applications for fellowship?
   1. **No, ERAS is not used** for applications. For Hematopathology Fellowship positions, only the selection process (the Match) is being handled by a central entity (the NRMP). Each program will continue to have their own application requirements, including their application forms and required letters of reference. Many programs utilize the CAP standard fellowship application:

[standardized-pathology-fellowship-application.pdf (cap.org)](https://documents.cap.org/documents/standardized-pathology-fellowship-application.pdf)

1. When can our program make an account with the NRMP and register for the Match?
   1. You can make an account **starting February 5^th^ 2025 at noon, ET**, which is when your program signs a legally binding Match Participation Agreement with the NRMP.
2. How does our program make an account with the NRMP and register for the Match?
   1. Follow this checklist, which includes a link to a video on “Institution and Program Match Process” and other resources: <http://www.nrmp.org/wp-content/uploads/2021/08/SMS-Program-Checklist.pdf>
   2. Since this is the first time Hematopathology fellowships are participating in the match, for this match only the Society for Hematopathology will facilitate creating a record for each participating hematopathology fellowship program with the NRMP. The SH will be in touch with the participating hematopathology fellowship PD to collect information that will be forwarded to the NRMP. The NRMP will use this information to create a record in the “Registration, Ranking, and Results” (R3) system; the NRMP will then send each program a token link by email when the Match opens on February 5th that you will use to register for the Match. Please respond promptly to requests for information from the SH-education committee in order to facilitate this process.
   3. *After the Match has opened and in subsequent years*, changes in program directorship or addition of new participating programs will be communicated directly with the NRMP.
3. Will applicants be able to apply to non-Match programs?
   1. Applicants can still apply to and interview at non-Match programs. However, they must decide whether or not to accept a position at a non-Match program **before certification within the Match** (before **April 16, 2025**). The Match is a binding commitment. If an applicant accepts a position at a non-Match program and does not withdraw from the NRMP Match, they will have committed a Match violation that may be disclosed to the medical school, current program director, designated institutional official, and other relevant parties.
4. May I ask applicants to disclose their interest in our program in terms of ranking preference?
   1. Programs **must not** request the applicants’ ranking preference or intentions. **However,** applicants **may voluntarily disclose their ranking preference or interest** to you during the application process. Likewise, **you may voluntarily disclose your ranking preference or interest** to applicants but applicants are **not permitted to request** your ranking preference from you.

1. What if an applicant who matched at our program does not want to, or cannot enroll?
   1. All programs and applicants must respect the binding nature of a match commitment and be prepared to honor the commitment if a match occurs with any program placed on a rank order list. If an applicant cannot or will not honor the commitment, a waiver or deferral must be requested from the NRMP (see link below). Waivers are granted only in cases of ineligibility, change of specialty, or unanticipated, serious, and extreme hardship.
2. Do we have to include all of our fellowship positions in the Match?

a. Yes. In order to have sufficient participant for a robust Match, participating hematopathology fellowship programs have agreed to include all of their fellowship positions in the Match.

1. What happens if our program commits a Match violation?
   1. Match violations are investigated by the NRMP and serious consequences can occur. These consequences can include the violations being reported to relevant entities, and being barred from future Matches. See also the NRMP Violations policy section 6b below:
      1. <https://www.nrmp.org/wp-content/uploads/2023/08/Violations-Policy_Ver.Aug2023.pdf>
2. Where can I find more information on the Match Code of Conduct?
   1. <https://www.nrmp.org/wp-content/uploads/2023/08/NRMP-Match-Codes-of-Conduct_Applicants.pdf>
   2. <https://www.nrmp.org/wp-content/uploads/2023/08/NRMP-Match-Code-of-Conduct_Programs.pdf>
3. What should I do if I feel a Match Code of Conduct violation has occurred?
   1. See the below website for reporting information: <https://www.nrmp.org/policy/reporting-and-investigation-of-violations/>
4. How does the Match work?
   1. <https://youtu.be/cnVe_NYIVAE> or scan this:
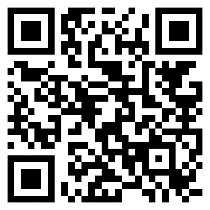


You can also view how the algorithm works at https://www.nrmp.org/intro-to-the-match/how-matching-algorithm-works/.

1. What if our fellowship program does not fill all positions?
   1. After Match Day, all programs in the Match with unfilled positions receive a list of applicants who did not Match. Applicants also receive a list of programs that did not fill. At this point, applicants can reach out directly to those programs to apply for and interview at these unfilled positions **outside of the NRMP Match**.
2. What are the benefits of using the NRMP Match?
   1. The NRMP Match ensures professional standards in conduct are used in the selection of fellows. The Match will help make the Hematopathology Fellowship selection process more equitable and timely.
3. Where can I find a list of hematopathology fellowships participating in the match?

a. <https://www.society-for-hematopathology.org/web/education-match.php>

b. The SH will be able to link from this webpage to your personal fellowship website.

c. The SH will promote this webpage through their social media.

1. How much does it cost for programs to register with the NRMP Match?

a. The Institution registration fee will be $250 per Match (price going to $300 subsequent year). Because the Institution fee is per Match, if your institution already has a Forensic Pathology fellowship participating in the Pathology Fellowship Match, there will be no additional institution registration fee for your participation.

The Program registration fee will be $60 per program track (price going to $100 for subsequent year). There will be a $60 fee per applicant matched to the program (price going to $75 for subsequent year). For more information: [Match Fees | NRMP](https://www.nrmp.org/intro-to-the-match/match-fees/)

1. Where can I find more information on the Match for Hematopathology?
   1. Society for Hematopathology webpage
   2. <https://www.nrmp.org/fellowship-applicants/>
   3. [info@society-for-hematopathology.org](mailto:info@society-for-hematopathology.org)
2. What do I tell an applicant who applies for a 2026-2027 position in my program?
   1. There is an FAQ document written for specifically for applicants on the SH website. There are also resources available on NRMP website (see answer to question 17).
   2. Here is a possible response: “We are excited to participate in the inaugural Hematopathology Fellowship NRMP Match for the 2026-2027 appointment year. A list of all the programs participating in the Match can be found on the SH website. An FAQ document on the match process written for applicants is also available on that website. Please send us your application materials including {insert program specific information here}. We will be in touch to schedule interviews, which will take place starting January 2025. You will need to register for the Pathology Fellowship Match in February 2025 with the NRMP (<https://www.nrmp.org/fellowship-applicants/> ) and can rank programs participating in the match with which you have interviewed.”
3. What do I do if I have questions that are specific to my hematopathology fellowship program?

a. You can reach out directly to the NRMP for more information.

b. Email: support@nrmp.org Toll Free: (866)653-NRMP Phone: (202)400-2233

1. How do I confirm the identity of the applicant(s) I want to rank within the NRMP system? (i.e. what if there are two people named John Smith in the system?)

a. Programs have the ability to search for and rank applicants using their AAMC ID, first and last name, and medical school in addition to their NRMP ID.

1. Is it possible to have a combined fellowship using the NRMP Match? (for example HP and clinical informatics or HP and MGP)

a. Yes. There are multiple options for doing this. Please reach out to the NRMP or to [info@society-for-hematopathology.org](mailto:info@society-for-hematopathology.org) for details on this process.
