# Supplementary material for: Joining the National Resident Matching Program Fellowship Match: the hematopathology experience
Source: Acad Pathol. 2025 Dec 12;13(1):100230. doi: 10.1016/j.acpath.2025.100230 (PMC12756700; doi:10.1016/j.acpath.2025.100230)
Supplement: Multimedia component 2 [file mmc2.docx]

**Supplemental Material 2**

Frequently Asked Questions: The NRMP Match for Hematopathology Fellowship

The selection process for prospective fellows in Hematopathology is switching to the NRMP Pathology Fellowship Match **for positions that begin in 2026**. This document assumes you are a prospective fellow who is applying for positions beginning in 2026. Hematopathology will participate in a multi-specialty Pathology Fellowship Match with Forensic Pathology and possibly other subspecialties in Pathology who are considering a Match.

1. When should I apply for fellowship positions?
   1. Your applications should be submitted to programs of your choice before **January 1 2025.** You are encouraged to submit applications well in advance of January 1, 2025. Note that you can start applying to positions before registering for the NRMP Match (see below).
2. When do interviews begin?
   1. Programs participating in the match have agreed to begin **interviewing starting in January 2025**.
3. Do I apply for positions through ERAS like I did for residency?
   1. **No, ERAS is not used** for applications. In residency, you used ERAS to apply for positions and the NRMP to match into them. For Hematopathology Fellowship positions, only the selection process (the Match) is being handled by a central entity (the NRMP). Refer to each program’s individual website for application requirements including things like application forms and required letters of reference. Many programs utilize the CAP standard fellowship application: [standardized-pathology-fellowship-application.pdf (cap.org)](https://documents.cap.org/documents/standardized-pathology-fellowship-application.pdf)
4. When can I make an account and register for the Pathology Fellowship Match?
   1. You can make an account **starting February 5^th^ 2025**.
5. How do I make an account and register for the Pathology Fellowship Match?
   1. Follow this step by step guide: <https://www.nrmp.org/wp-content/uploads/2021/08/Registering_for_SMS_Match-App.pdf>
6. What items/documents are required to register for the Match?
   1. You will need your USMLE ID, NBOME, and/or AOA ID, USMLE or COMLEX Test Scores, and AAMC ID.
7. When can I enter my rank list of programs?
   1. Ranking opens on **March 12, 2025 at noon, ET.**
8. When can I no longer edit my rank list?
   1. Ranking certification must be completed by **April 16, 2025 at 9:00 PM, ET.**
9. When is Match Day?
   1. **Match day is April 30, 2025 at noon, ET.**
10. Can I apply to non-Match programs?
    1. You may apply to and interview at non-Match programs. However, you must decide whether or not to accept a position at a non-Match program **before certification within the Match** (before **April 16, 2025**). The Match is a binding commitment. If you accept a position at a non-Match program and do not withdraw from the NRMP Match, you have committed a Match violation that may be disclosed to your medical school, current program director, designated institutional official, and other relevant parties.
11. May I ask programs to disclose their interest in me in terms of ranking preference?
    1. Applicants **must not** request the program’s ranking preference or intentions. **However,** programs **may voluntarily disclose their ranking preference or interest** to you during the application process. Likewise, **you may voluntarily disclose your ranking preference** or interest to programs but programs are **not permitted to request** your ranking preference from you.
12. How should I rank programs?
    1. Applicants should create rank order lists based on their true preferences, the characteristics of the programs interviewed, and the perceived alignment of the applicant’s capabilities and interests with program mission, aims, and eligibility. Applicants must respect the binding nature of a match commitment and be prepared to honor the commitment if a match occurs with any program placed on a certified rank order list.
13. What if I match to a program and don’t want to go there?
    1. Only rank programs you would WANT to attend. If you Match to a program, you have created a binding commitment to attend that program. If you cannot or will not honor the commitment, a waiver or deferral must be requested from the NRMP (see link below). Waivers are granted only in cases of ineligibility, change of specialty, or unanticipated, serious, and extreme hardship.
14. What happens if I commit a Match violation?
    1. Match violations are investigated by the NRMP and serious consequences can occur. These consequences can include being barred from future Matches, and programs (even if they are not in the Match) may be barred from giving you an offer. See also the NRMP Violations policy section 6 below:
       1. https://www.nrmp.org/wp-content/uploads/2023/08/Violations-Policy_Ver.Aug2023.pdf
15. Where can I find more information on the Match Code of Conduct?
    1. <https://www.nrmp.org/wp-content/uploads/2023/08/NRMP-Match-Codes-of-Conduct_Applicants.pdf>
    2. <https://www.nrmp.org/wp-content/uploads/2023/08/NRMP-Match-Code-of-Conduct_Programs.pdf>
16. What should I do if I feel a Match Code of Conduct violation has occurred?
    1. See the below website for reporting information: <https://www.nrmp.org/policy/reporting-and-investigation-of-violations/>
17. How does the Match work?
    1. <https://youtu.be/cnVe_NYIVAE> or scan this:
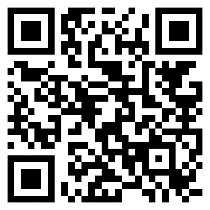


You can also view how the algorithm works at https://www.nrmp.org/intro-to-the-match/how-matching-algorithm-works/.

1. What if I do not Match?
   1. After Match Day, all programs in the Match with unfilled positions receive a list of applicants who did not Match. Applicants also receive a list of programs that did not fill. At this point, you may reach out directly to these programs to apply for and interview at these unfilled positions **outside of the NRMP Match**.
2. What are the benefits of using the NRMP Match?
   1. The NRMP Match ensures professional standards in conduct are used in the selection of fellows. The Match will help make the Hematopathology Fellowship selection process more equitable and timely.
3. Where can I find a list of hematopathology fellowships participating in the match?

a. <https://www.society-for-hematopathology.org/web/education-fellowship-match.php>

1. How much does it cost to register with the NRMP Match?

a. The standard registration fee for applicants is $70; this includes the listing of up to 20 unique program codes on the rank order list. For more information: [Match Fees | NRMP](https://www.nrmp.org/intro-to-the-match/match-fees/)

1. Where can I find more information on the Match for Hematopathology?
   1. <https://www.society-for-hematopathology.org/web/education-match.php>
   2. <https://www.nrmp.org/fellowship-applicants/>
   3. [info@society-for-hematopathology.org](mailto:info@society-for-hematopathology.org)
